# Genome-wide characterization of NAC transcription factors in *Camellia sinensis* and the involvement of CsNAC28 in drought tolerance

**DOI:** 10.3389/fpls.2022.1065261

**Published:** 2022-11-24

**Authors:** Xueying Zhang, Linying Li, Zhuoliang Lang, Da Li, Yuqing He, Yao Zhao, Han Tao, Jiqian Wei, Qingsheng Li, Gaojie Hong

**Affiliations:** ^1^ Key Laboratory for Managing Biotic and Chemical Threats to the Quality and Safety of Agro-products, Key Laboratory of Biotechnology in Plant Protection of Ministry of Agriculture and Rural Affairs, Key Laboratory of Biotechnology in Plant Protection of Zhejiang Province, Institute of Virology and Biotechnology, Zhejiang Academy of Agricultural Sciences, Hangzhou, China; ^2^ College of Advanced Agricultural Sciences, Zhejiang A&F University, Hangzhou, China; ^3^ Institute of Sericulture and Tea, Zhejiang Academy of Agricultural Sciences, Hangzhou, China; ^4^ Ecology and Energy Section, Hangzhou Agricultural Technology Extension Center, Hangzhou, China

**Keywords:** *Camellia sinensis*, expression pattern, NAC transcription factor, drought stress, abscisic acid

## Abstract

The NAM, ATAF1/2, and CUC2 (NAC) transcription factors, which are members of a plant-specific gene family, play critical roles during the growth and development of plants and in their adaption to environmental stress. Few NAC transcription factors have been functionally characterized in tea plants (*Camellia sinensis*). Based on the analysis of the gene structure, motif pattern, and evolutionary relationship, we identified 104 NAC genes in *C. sinensis*. Among them, *CsNAC28* is constitutively expressed in all organs, and most significantly, exhibiting remarkable responsiveness to abscisic acid (ABA) treatment and drought stress. ABA is a primary stress-related hormone. Recently, ABA-responsive element binding factor 2 (*CsABF2*) was identified in the ABA pathway of *C. sinensis.* However, the involvement of the *CsABF2*-mediated ABA pathway in regulating CsNACs was not known. Herein, a series of biochemical and genetic approaches supported the fact that *CsNAC28* could potentially act as a transcription factor in the downstream of *CsABF2*. Furthermore, we investigated the function of *CsNAC28* in the adapting of a plant to drought stress. The results showed that overexpression of *CsNAC28* in Arabidopsis conferred hypersensitivity to ABA treatment and decreased the accumulation of reactive oxygen species (ROS), resulting in improved dehydration tolerance. Under conditions of drought, the expression levels of ABA pathway-related genes and drought stress‒inducible genes were greater in *CsNAC28* overexpression lines than in the wild type. Our study’s comprehensive characterization of NAC genes in *C. sinensis* could serve as a foundation for exploring the molecular mechanism of CsNAC-mediated drought responsiveness.

## Introduction

The tea plant *C. sinensis* is a major crop harvested for tea that is consumed worldwide, and it has been widely cultivated in tropical and subtropical areas (Liu et al., 2015). Its abundance of secondary metabolites contribute to the nutrients, a clean taste, and rich flavors beneficial to human health (Yang and Hong, 2013; Zhao et al., 2020). In recent years, the increasing frequency of extreme temperatures and drought stress affected the yield and quality of tea leaves (Wang et al., 2016a). Transcription factors play critical roles in activating gene transcripts through binding to the target gene promoter regions and regulating the plant growth, development, and biotic and abiotic stress processes (Pandey and Shukla, 2015).

NAC (NAM-ATAF1/2-CUC) transcription factors constitute one of the most diverse transcription factor families in plants; they contain highly conserved N-terminal DNA binding domains (150 amino acids) but relatively variable C-terminal regions (Souer et al., 1996; Ooka et al., 2003; Ernst et al., 2004). The N-terminal areas contain five subdomains (A, B, C, D and E), such as NAM (no apical meristem) in a petunia mutant and ATAF1/2 and CUC2 (cup-shaped cotyledon) in Arabidopsis. Subdomains B and E are divergent and may be associated with the NAC gene’s functional diversity, whereas subdomains A, C, and D are generally conserved (Olsen et al., 2005). The C-terminal regions are responsible for activating or repressing the expression of downstream genes (Kjaersgaard et al., 2011; Lindemose et al., 2014). Additionally, some NAC transcription factors have transmembrane motifs, which function as endoplasmic or plasma membrane anchors at the C-terminal (Seo et al., 2008; Li et al., 2016).

The NAC transcription factors regulate many critical biological processes that are commonly associated with stress resistance, and they have been extensively identified in Arabidopsis (105), rice (138), maize (147), sunflowers (151), tomatoes (93), bananas (167), peppers (104), *Populus trichocarpa* (163), and apples (180) because of the availability of their whole-genome sequences (Ooka et al., 2003; Hu et al., 2010; Nuruzzaman et al., 2010; Su et al., 2013; Cenci et al., 2014; Diao et al., 2018; Jin et al., 2020; Wang et al., 2020; Bengoa Luoni et al., 2021). Many studies have demonstrated that NAC transcription factors increase resistance to drought by activating downstream genes involved in the ABA pathway. In Arabidopsis, ATAF1, ATAF2, ANAC019, ANAC055, RD26/ANAC072, and ANAC096 contribute to drought tolerance through an ABA-mediated pathway (Fujita et al., 2004; Tran et al., 2004; Jiang et al., 2009; Jensen et al., 2010; Xu et al., 2013; Liu et al., 2016; Jiang et al., 2019). Overexpression of OsNAC5, OsNAC6, and OsNAC9 increases the sensitivity to ABA and results in enhanced tolerance to drought in rice (Redillas et al., 2012; Lee et al., 2017; Bang et al., 2022). In wheat, TaSNAC8-6A, TaASNAC4-3A, and TaNAC069 could activate the expression of drought-related genes (Xue et al., 2011; Mao et al., 2020; Mei et al., 2021). In maize, ZmSNAC1, ZmNAC33, ZmNAC49, ZmNAC55, ZmNAC84, and ZmNAC111 drive drought responses through the ABA pathway (Lu et al., 2012; Mao et al., 2015; Mao et al., 2016; Liu et al., 2019; Han et al., 2021; Xiang et al., 2021). In the tea plant, the involvement of CsNAC in responding to drought stress has not been determined.

The high-quality genome sequences of tea plants have been published, and, therefore, it was possible to identify 104 CsNACs by scanning the genome at the chromosome level (Wei et al., 2018). We analyzed the gene structure, motif pattern, chromosome distribution, synteny, and evolutionary relationship among Arabidopsis, *P. trichocarpa*, and *C. sinensis*. To investigate the involvement of CsNACs in the adaption to drought stress, we chose *CsNAC28* as a candidate. The results showed that *CsNAC28* localized in the nucleus and possessed transactivation activity. Furthermore, the overexpression of *CsNAC28* in Arabidopsis enhanced ABA sensitivity and upregulated the expression of drought-tolerance-related genes, thus improving the dehydration tolerance. This work improved our understanding of CsNAC transcription factors and the function of NACs in the adaption to drought stress, thereby contributing to tea plant breeding programs.

## Materials and methods

### Identification and structure analysis of NAC genes in *C. sinensis*

To identify the tea plant (*C. sinensis* cv. ShuChaZao) NAC genes, the hidden Markov model (HMM) profile of the NAC domain (PF00170) was downloaded from Pfam (http://pfam.xfam.org/) and the NAC protein sequences were searched with an e-value cutoff of 1e^-5^. Then, to determine their presence and completeness of the NAC domain, all of the putative NAC genes were identified manually one by one (E value<1.0) in INTERPORSCAN and SMART (http://smart.embl-heidelberg.de/). Molecular weight, isoelectric points, subcellular localization, and transmembrane helices of candidate NAC proteins were detected in ExPASy (http://expasy.org/tools/), WoLF PSORT (http://wolfpsort.org/) and TMHMM (http://www.cbs.dtu.dk/services/TMHMM/) server v2.0.

### Phylogenetic, gene structure and motifs composition of CsNACs

The NAC sequences of coding proteins from Arabidopsis, *Oryza sativa* and *C. sinensis* were aligned using ClustalX 2.0. A phylogenetic tree consisting of 345 NACs was constructed by MEGA7.0 using the neighbor-joining (NJ) tree with default parameters. 1000 replicates were used to produce bootstrap values. The exon/intron structure of NAC genes was displayed by the Gene Structure Display Server program (GSDS http://gsds.cbi.pku.edu.cn/) platform, and the NAC conserved motif was characterized by MEME, with a cap of motifs set to 10.

### Chromosomal mapping and gene duplication analysis

The NAC genes were mapped by MapInspect (http://www.plantbreeding.wur.nl/UK/software_mapinspect.html) with the *C. sinensis* genome database. The gene duplication events of NAC genes were examined by MCScanX software with default parameters. To visualize the duplicated regions in the *C. sinensis* genome, the Circos-0.67 program (http://circos.ca/) was used to draw between matching genes. The homology of the NAC genes between *C. sinensis* and the other species (*Arabidopsis thaliana*, *Oryza sativa and Populus trichocarpa*) was analyzed by Dual Synteny Plotter of TBtools (Chen et al., 2020).

### NAC transcription factors expression pattern in *C. sinensis*

The expression pattern data from distinct tissues (Root, Stem, Old leaf, Mature leaf, Young leaf, Apical bud, Folwer and Fruit) have been previously reported in the genome sequencing research of ‘ShuChaZao’, which was downloaded from http://tpia.teaplant.org/index.html (Wei et al., 2018). The NAC genes expression level were evaluated using fragments per kilobase per million reads mapped (FPKM) and the data were displayed as Log_10_^(FPKM value)^ in a heat map using the Mev4.9.0 software. The leaves of tea plant (*C. sinensis* vs LongJing43) were sprayed with 100 µM ABA for ABA treatments (ddH_2_O as control). The roots of tea plant seedlings were irrigated with 20% PEG for drought treatments (ddH_2_O as a mock control). The real-time PCR primers for the CsNACs of interest were designed by Beacon Designer 7.0 software (**Supplementary Table S1**). Glyceraldehyde-3-phosphate dehydrogenase (GAPDH, accession number: KA295375.1) was employed as an internal reference. SYBR Green (Roche, Basel, Switzerland) was used in real-time PCR on an ABI7900HT Sequence Detection System (Applied Biosystems, Waltham, MA, USA).

### Transcriptional self-activating of NACs proteins and subcellular localization

The cDNA of CsNAC28 were inserted into the pGBK vector digested with BamH I and Nde I. pGBK-CsNAC28 and pGBK-Lam were transformed into the yeast strain Y_2_H and plated on the SD/-Trp plates individually. Colonies were further transferred to SD/-Trp/ABA (250ng) medium for 3-5 days at 30°C.

The binary vector PCV-eGFP -N1 was digested with Apa I enzyme before the cDNA of CsNAC28 was inserted. PCV-CsNAC28-eGFP-N1 and PCV-eGFP-N1 vectors were introduced into *Agrobacterium tumefaciens* strain GV3101, then transiently transformed into tobacco. The GFP fluorescence was imaged at 495-545 nm using a Leica TCS SP5 (Leica Microsystems, Bannockburn, IL, USA) confocal laser-scanning microscope after infiltrating 44-48h.

### Yeast one-hybrid assay

The cDNA of CsABF2 was inserted into a pGAD vector digested with BamH I and Nde I. The *CsNAC28* promoter was inserted into the pABAi vector digested with Hind III and Sal I. CsNAC28p-pABAi were linearized with Bstb I, then transformed into Y1H strain and plated on SD/-Ura medium. Colonies containing the CsNAC28 promoter were used to generate receptor states, which were then transformed with the CsABF2- pGAD (pGAD plasmid as a control) and plated into SD/-Leu medium. Colonies were further transferred to SD/-Leu medium with 300ng/ml ABA for three days.

### Dual-luciferase assay for CsNAC28

Dual-luciferase assays were conducted in accordance with He et al. (2021). The pGreenII0800- LUC vector was digested with Pst I and Nco I and used to insert the 1388 bp promoter region of CsNAC28. The pCambia2300 vector was digested with Kpn I and Bam HI and used to insert the cDNA of CsABF2. Dual-Luciferase Reporter Assay kit (Promega, Madison, WI, USA) was used to measure the ratio of firefly luciferase to renilla luciferase of the CsNAC28 promoters with and without the effect of CsABF2.

### Transient gene suppression in tea plants

Gene suppression assays were carried out as described by Hu et al. (2022). *CsABF2* was used as input sequences to design candidate antisense oligonucleotides (AsODNs) with the Soligo software (Ding and Lawrence, 2003) (**Supplementary Table S1**). Both sense oligonucleotides (sODNs) and gene-specific AsODNs were infiltrated with at least ten individual tea plants. The leaves were harvested after 24 h treatment, quick-frozen in liquid nitrogen, and stored at -80°C.

### Plant phenotype under ABA and drought treatments

The cDNA of CsNAC28 was cloned into the pCambia1300 vector under the control of the 35s promoter, Transgenic plants of CsNAC28-Flag (three copies of the flag in tandem) overexpressing in Col-0 were generated. The *Agrobacterium* strains GV3101 containing CsNAC28-pCambia1300 were introduced into Arabidopsis plants by the *Agrobacterium tumefaciens*-mediated floral dip method (Clough and Bent, 1998). Hygromycin resistance was used to screen out of ten independent transgenic lines. T3 homozygous progenies of transgenic lines (OE1, OE2 and OE3) were chosen to be studied further. The sequence of primers used in this investigation is listed in **Supplementary Table S1**.

To conduct the germination experiment, the cleaned seeds were sown on 1/2MS solid medium (90 seeds were sown for each line), which contained ABA (0 and 5µM) and mannitol (0, 100 and 200 mM) in varying concentrations, respectively. Then the mediums were placed in a refrigerator at 4°C for three days to break seed dormancy and transferred into a greenhouse 22°C under long-day ling conditions (16h light/8h dark). The germination number and root length were recorded once daily. The 4-week-old seedlings were dehydrated for 14 days for drought stress treatment, and re-water. Each experiment was repeated three times (Chen et al., 2017).

### Measurement of reactive oxygen species

WT and transgenic plants were subjected to drought stress and three individual plant leaves were sampled. To determine the hydrogen peroxide (H_2_O_2_) and superoxide (O^2-^) content, the leaves were stained by 3,3’-diaminobenzidine (DAB) and nitroblue tetrazolium (NBT) according to previously described methods (Yang et al., 2018).

### Statistical analysis

All experiments were carried out with at least three independent biological replicates. Each measurement was carried out in triplicate. Data represent the mean ± sd of three biological replicates. Data were statistically analyzed by one-way analysis of variance (ANOVA) performed using SPSS.

## Results

### Identification of NACs in *C. sinensis*

The genome database of “*C. sinensis*” allows for the identification of NAC gene members in the tea plant (Wei et al., 2018). A total of 104 NACs was characterized with a conserved NAC domain (PF01849) or NAM domain (PF02365). The NACs’ amino acid residue counts ranged from 134 to 679, their putative molecular weights ranged from 15.37 to 77.14 KDa, and their isoelectric points (pIs) were 4.6 to 9.91. An examination of their chromosomal locations showed that 104 NACs were matched to the 15 chromosomes of the *C. sinensis* genome and that the number of NACs in each chromosome differed, ranging from 3 (chr15) to 15 (chr09) (**Supplementary Figure S1**). According to the chromosomal position, which was named CsNAC1 to CsNAC104 (**Supplementary Table S2**), the 104 NAC proteins were predicted to be located in the nucleus or cytoplasm. Additionally, eight NACs contained a transmembrane domain. *CsNAC20, CsNAC64, CsNAC74*, and *CsNAC99* had transmembrane domains at the C-terminal, *CsNAC55* had two transmembrane domains, and *CsNAC2, CsNAC68*, and *CsNAC100* had transmembrane domains at the N-terminal.

### Phylogenetic and gene structure analysis of CsNAC genes

**Figure 1:**
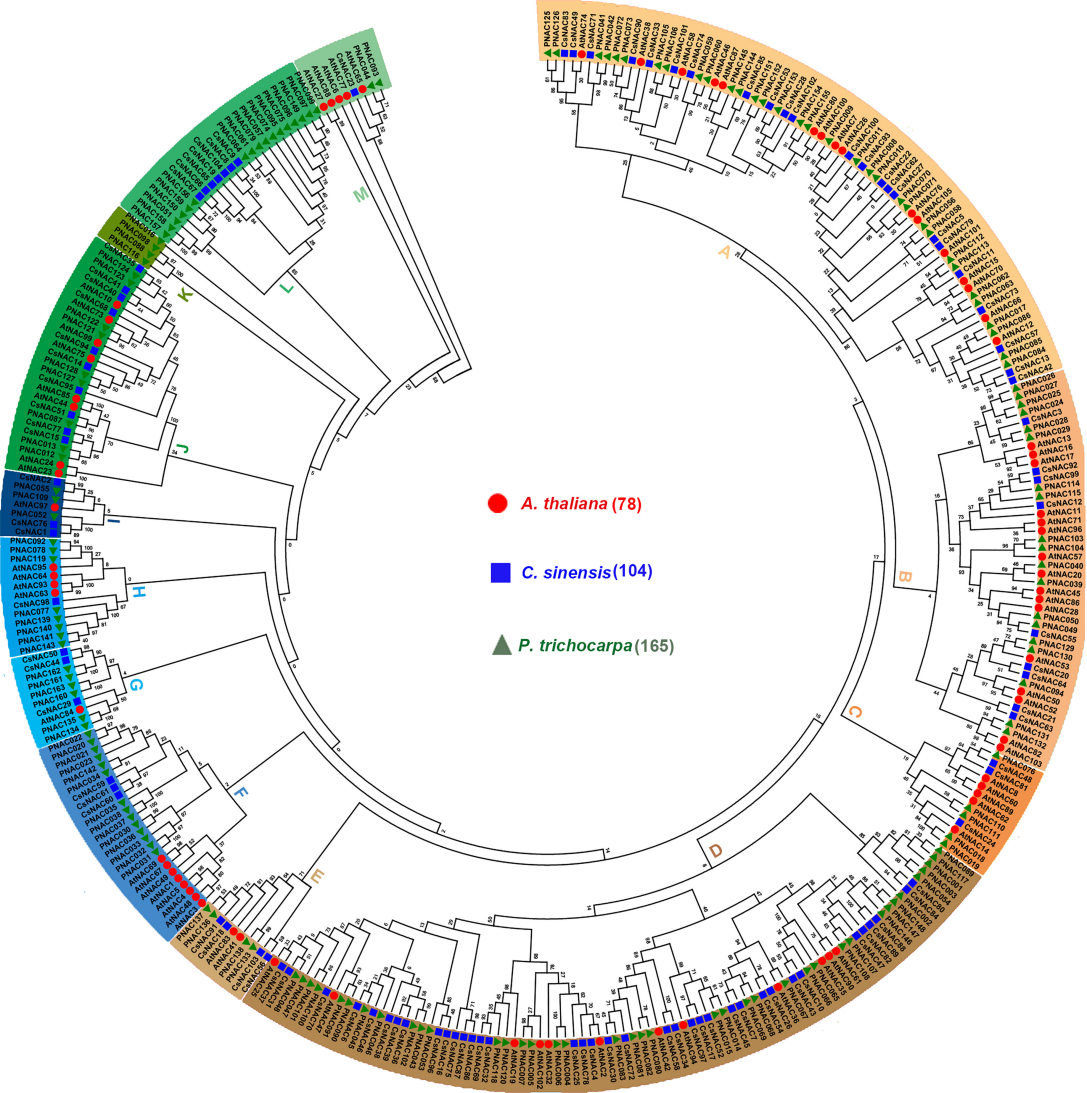
Phylogenetic tree of NACs from *Arabidopsis thaliana*, *Populus trichocarpa* and *C. sinensis*. Amino acid sequences were aligned using Clustal X software and subjected to phylogenetic analysis using MEGA X software by the NJ method with 1,000 bootstrap replicates.

To evaluate the phylogenetic relationship of the NAC proteins in *C. sinensis* and other species, an unrooted neighbor-joining tree was created with 345 NAC proteins from three plant species (i.e., 78 from Arabidopsis, 163 from *P. trichocarpa*, and 104 from *C. sinensis*). The results showed that 345 NAC proteins were grouped into 13 subfamilies, named subfamilies A to M. All of them were unevenly distributed in 11 subfamilies; subfamily L contained 17 PNACs and 7 CsNACs, whereas subfamily K contained only 4 PNACs but no AtNACs or CsNACs. Generally, the CsNAC proteins had closer relationships with the NACs from *P. trichocarpa* than those from Arabidopsis, and this was confirmed by the current plant evolutionary history and provided more useful references for functional identification in tea plants (**Figure 1**).

**Figure 2:**
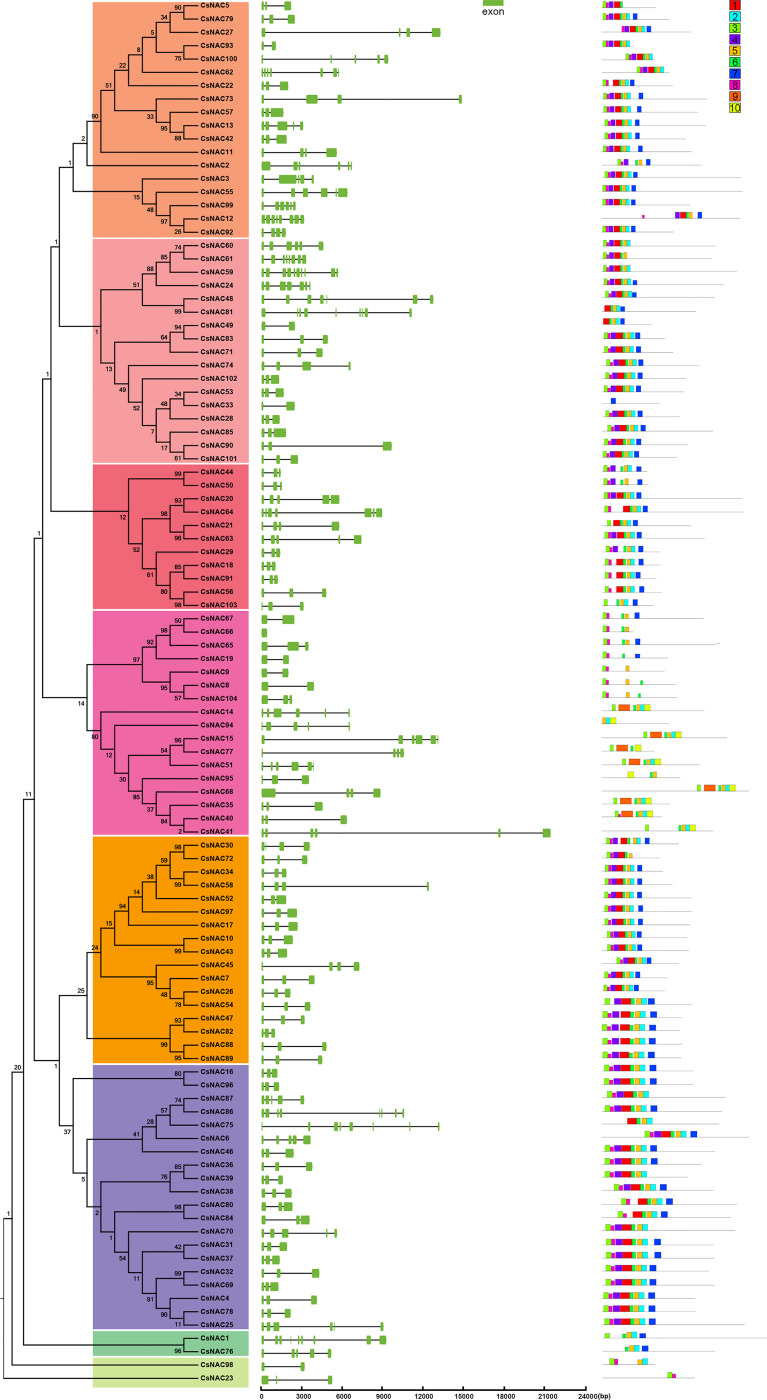
Gene structure and motif organization of *NAC* genes in *C. sinensis*. Intron and exon structures of NACs are graphically represented by black lines and orange boxes, respectively. The protein sequences of NACs was used to predict the conserved regions and motifs. Conserved motifs are indicated by a colored box numbered 1 to 10.

To further explore the evolution of the NAC gene family, we analyzed the structural features of the NAC genes in *C. sinensis*. *CsNAC41* has a gene structure that is longer than 20 kb, whereas *CsNAC66* has the shortest structure, at 405 bp. Of all the NAC genes, *CsNAC59* contained the most (10) exons. No introns were found in *CsNAC66;* over half (54, or approximately 51.9%) of the NAC genes had three exons; and most of the NAC genes shared a common exon/intron structure and had intron phases that were clustered in the same subgroup (**Figure 2**). In addition, 10 conserved motifs of the 104 NAC genes were investigated by the MEME program to explore genetic diversification in *C. sinensis*. The lengths of these conserved motifs ranged from 11 to 50 amino acids, with a highly diverse distribution. *CsNAC33* only contained one motif, whereas eight similarly ordered motifs (motifs 3, 8, 4, 1, 6, 5, 2, 7) were present in most NACs. Except for the fourth subgroup, the compositions of the conserved motifs and orders of the NAC protein sequences in the same group were similar. Motifs 9 and 10 were unique to subgroup four, suggesting that they had specific functions that benefited this group (**Figure 2**). An analysis of the exon/intron and motif compositions further showed that the genes that have developed in offspring may have functional redundancy.

### Syntenic analysis of NAC genes in *C. sinensis* and other species

Intrachromosomal and interchromosomal evolutionary research showed that 9 pairs of tandem duplicate events occurred in the same chromosome (tandem duplications were defined as genes on the same chromosome that were mapped at a distance ≤ 100 kb) and that 49 segmental duplication events occurred on all chromosomes, which suggested that the expansion of the CsNAC family was mainly the result of interchromosomal duplication events (**Supplementary Figure S2**).

**Figure 3:**
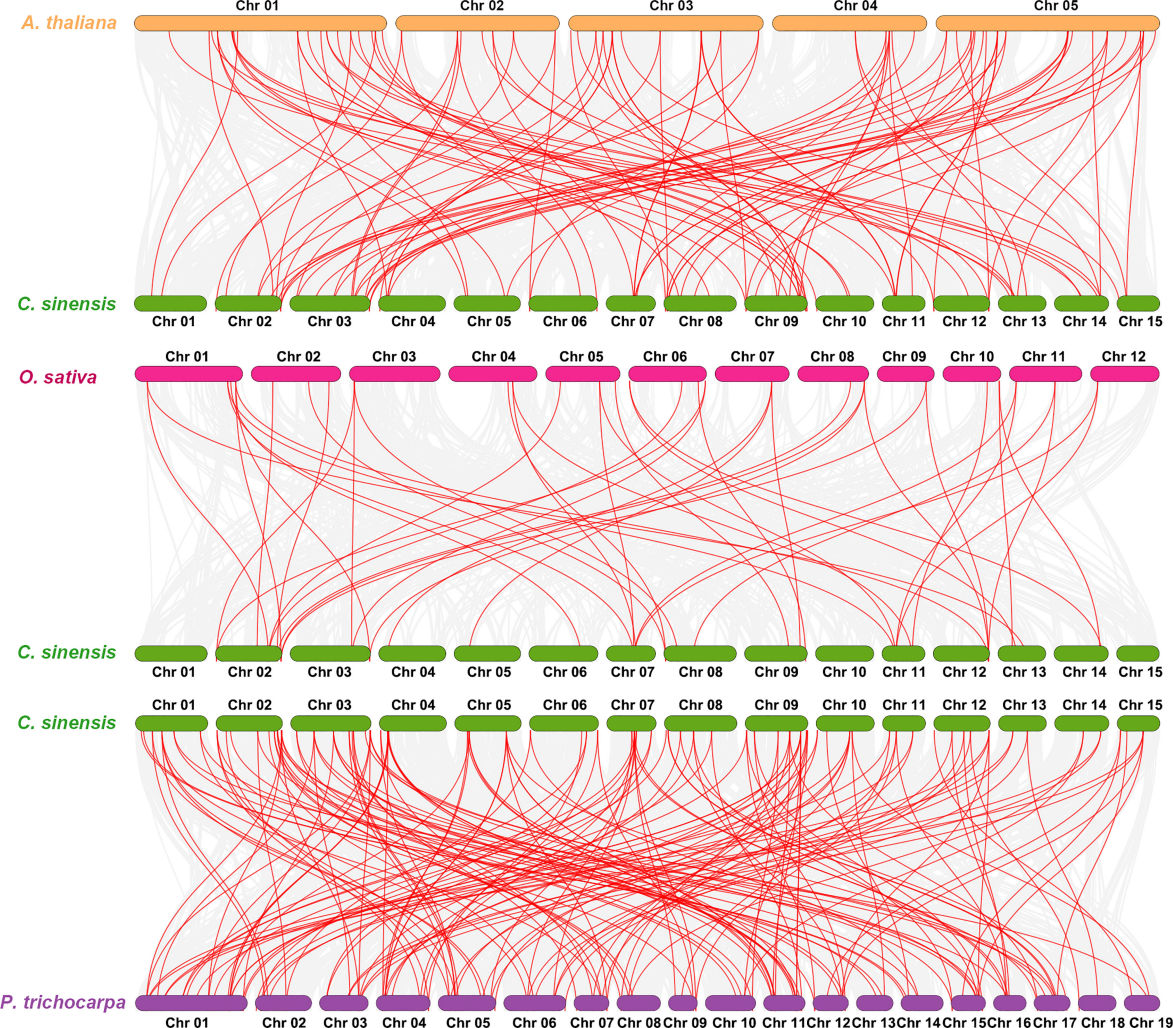
Synteny analysis of NAC genes between *C. sinensis* and three representative species. Red lines highlight the syntenic NAC gene pairs with the *Camellia sinensis* and other plant genomes, whereas gray lines in the background indicate the collinear blocks.

A collinearity assay of the NAC gene family in *C. sinensis*, Arabidopsis, *Oryza sativa*, and *P. trichocarpa* was carried out to explore the species’ evolutionary relationships, and it found 101, 45, and 165 homologous pairs between *C. sinensis* and the other three species, respectively. The results showed that *C. sinensis* and *P. trichocarpa* had more homologous genes, which was consistent with their phylogenetic relationship (**Figure 3**).

### Tissue-specific expression profiles of NACs in *C. sinensis*

**Figure 4:**
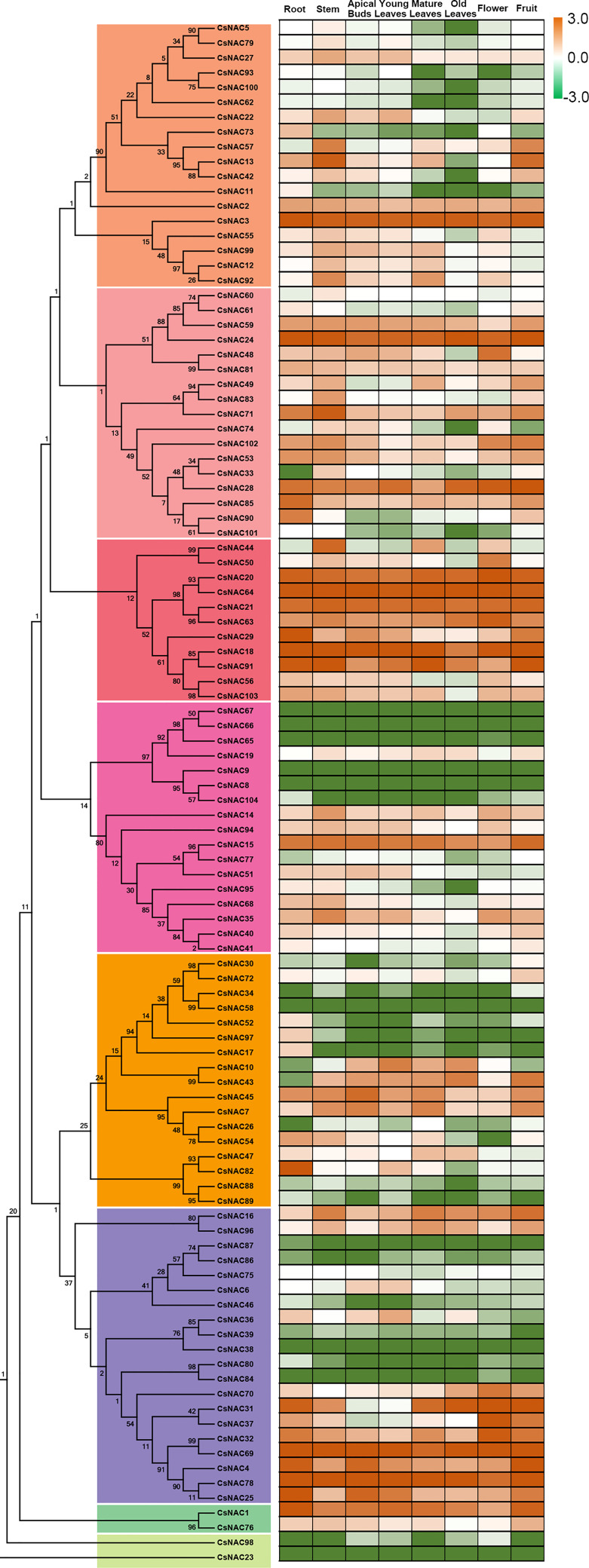
Expression patterns of CsNACs in different tissues of *C. sinensis*. The expression patterns of CsNACs genes in eight tissues (Root, Stem, Old leaf: germinated in previous years; Mature leaf: geminated in the spring and harvested in the autumn; Young leaf: the first and second leaves follow the apical bud; Apical bud: unopened leaves on the top of activity growing shoots, Flower and Fruit) of tea plant were calculated using Log_10_^(FPKM)^. The most of the data were distributed between -3 and +3, which indicated high and low expression levels.

Cultivated tea tree species differ greatly in terms of plant morphology and economic characteristics. We determined the abundance of NAC transcripts in various tissues, including the apical bud, young leaf, mature leaf, flower, fruit, old leaf, stem, and root tissue of *C. sinensis* cv. ShuChaZao, to explore the tissue specificity. As shown in **Figure 4**, the expression levels of 16 *CsNACs* (*CsNAC8/9/23/34/38/39/46/58/65/66/67/80/86/87/84*/*98*) were very low or undetectable in all tested tissues, possibly due to the fact that they were pseudogenes; 88 CsNAC members were expressed in at least one of the organs of *C. sinensis*; and 14 *CsNACs* (*CsNAC1/3/4/15/18/20/21/24/28/63/64/69/78*/*91*) were predominantly expressed in all tissues with a Log_10_^(FPKM value)^ of greater than 1. Ten *CsNACs* (*CsNAC7/10/25/29/35/36/43/45/48*/*59*) were highly expressed in buds and leaves. The transcripts of 18 CsNACs were found in the roots, and the transcripts of 20 CsNACs were found in the flowers or fruits (**Figure 4**).

### AREB *cis-*acting elements of CsNAC gene promoter regions

ABA-responsive element-binding factors (AREB/ABFs) are master regulators of the transcriptional response to ABA. Most of them activate the expression of drought-responsive genes *via* the direct binding of the ABA-responsive element (ABRE: PyACGTGG/TC) to improving drought tolerance (Fujita et al., 2011). Thus, we analyzed a sequence 2 kb upstream from the translation initiation site of the CsNACs. The results showed that 78 of the CsNAC promoters contained at least one ABRE element. The number of ABRE elements ranged from one to eight. Specifically, 27 of the CsNAC promoters had one ABRE element, 17 contained two ABRE elements, and 11 contained three or five ABRE elements. The other CsNAC promoters contained four, six, or seven ABRE elements, and only *CsNAC78* contained eight ABRE elements (**Supplementary Figure S3A**). To further examine the potential role of CsNACs in the response to drought stress, transcriptome data for tea plants under drought stress treatment were acquired from the Tea Plant Information Archive (http://tpdb.shengxin.ren/index.html). The expression patterns of 50 CsNAC genes in response to drought stress are shown in **Supplementary Figure S3B**. The expression of 20 of these CsNACs was induced significantly, and the expression of 15 of these was high after 24 hours of drought treatment.

**Figure 5:**
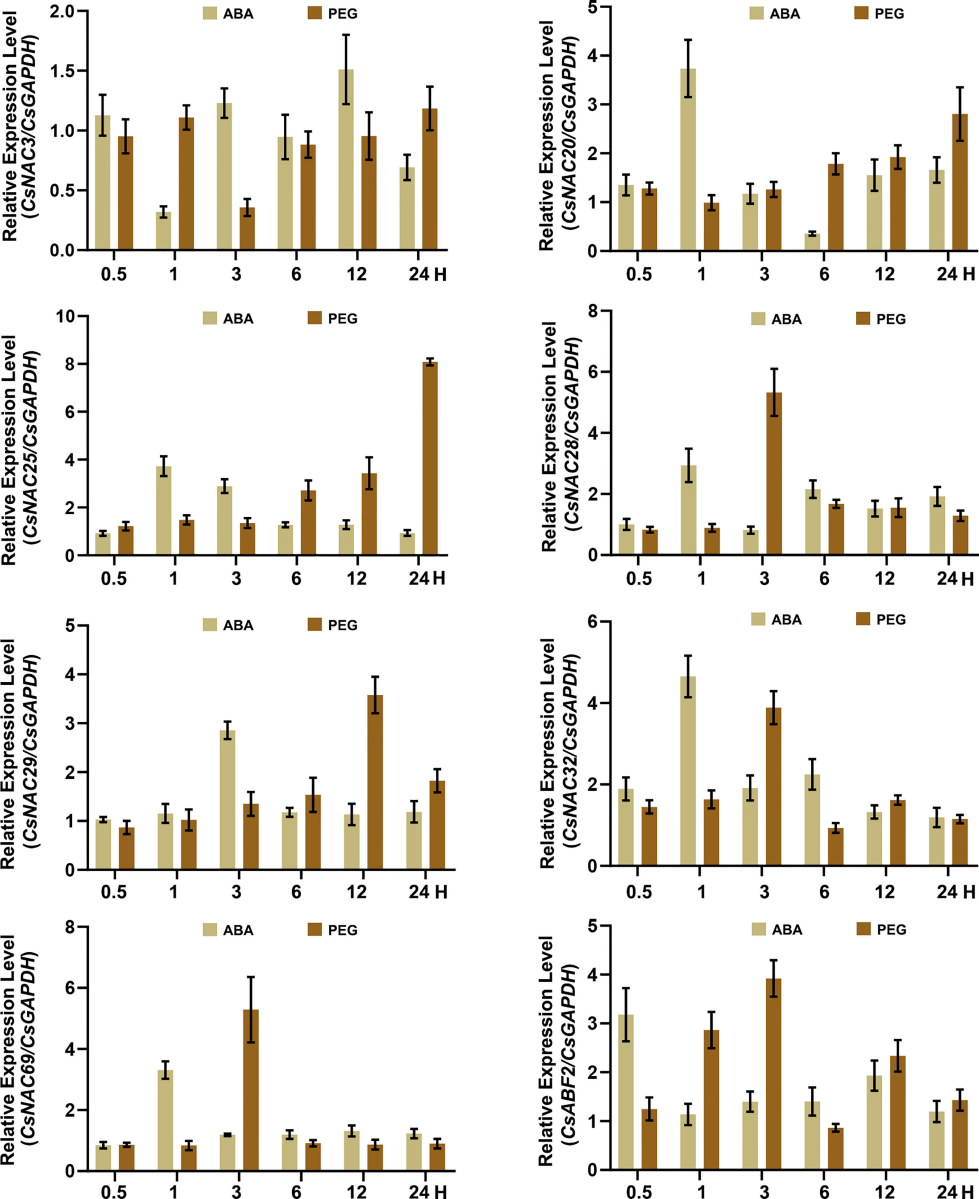
Expression patterns of CsNACs in *C. sinensis* under ABA and PEG. Error bars indicate SD of three biological replicates.

Considering the expression pattern of the CsNACs and the existence of ABRE elements in their promoters, seven CsNACs were selected for attempts to detect their responsiveness to ABA treatment and drought stress through the quantitative real-time polymerase chain reaction (qRT-PCR). In most of the selected candidate genes, expression was significantly induced after exposure to ABA treatment and drought stress. The expression levels of *CsNAC20*, *CsNAC25*, *CsNAC28*, *CsNAC32*, and *CsNAC69* were upregulated two-fold to four-fold at 1 hour (H), *CsNAC25* were induced after 3 H of ABA treatment. The gene expression of most CsNACs had similar profiles for ABA treatment and drought stress, although drought stress exhibited a certain lag effect on the induction of expression of CsNACs. To be specific, the expression of *CsNAC20* and *CsNAC25* was upregulated three-fold and nine-fold at 24 H, respectively; that of *CsNAC28*, *CsNAC32*, and *CsNAC69* was significantly increased at 3 H; and that of *CsNAC29* was upregulated at 12 H. The expression of *CsNAC3* was repressed by ABA and drought stress, and the peak values of relative expression appeared at 1 H and 3 H, respectively (**Figure 5**). The results showed that the expression of most NACs was induced by ABA treatment and drought stress.

### Subcellular localization and transcriptional activation ability of CsNAC proteins

**Figure 6:**
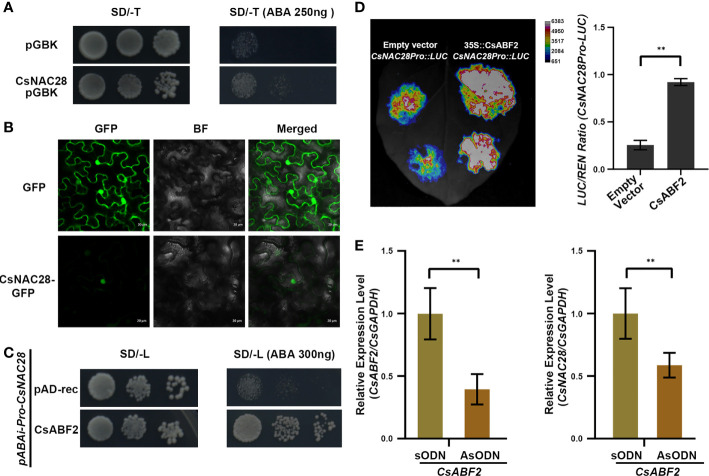
The potential function of CsNAC28 in tea plant. **(A)** Transactivation analyses of CsNAC28 in yeast. Negative control, and the fusion constructs were transformed into the Y_2_H strain and successively incubated in SD/-Trp media and SD-Trp/ABA (250ng) plate. **(B)** Subcellular localization of CsNAC28. GFP and CsNAC28-GFP were transiently expressed in tobacco leaves. GFP, Green fluorescence image; BF, Bright-field microscopy image; Merge, Merged bright-field and green fluorescence images. **(C)** Yeast one-hybrid assay to confirm binding of CsABF2 to the *CsNAC28* promoter. **(D)** Relative LUC/REN ratio from transient expression assays of the *CsNAC28* promoter in the present of CsABF2. Error bars indicate the SD of three biological replicates. **(E)** *CsABF2* and *CsNAC28* expression in control (*CsABF2/*sODN) and *CsABF2/AsODN* tea leaves. Error bars indicate the SD of three biological replicates. **Student’s test, P < 0.01.

Both *CsNAC28* and *CsNAC69* were constitutively expressed in all organs, and their expression was further induced by ABA treatment and drought stress. Because *CsNAC69* is the homologue of *ANAC019*, which confers drought tolerance in Arabidopsis (Jensen et al., 2010), we investigated whether *CsNAC28* participated in the drought stress response in tea. The full-length *CsNAC28* was fused into the DNA binding domain to investigate the transcriptional activation of *CsNAC28*. Each CsNAC-pGBK-pGAD pair was individually co-transformed into the yeast cells Y_2_H and further selected on a quadruple dropout medium. As shown in **Figure 6A**, the negative control did not grow but the *CsNAC28* transformant grew well, indicating that *CsNAC28* acts as a transcription factor with transcriptional activity in yeast strains.

To explore the sub-localization of *CsNAC28*, *Agrobacterium tumefacient* strains GV3101 containing either a GFP empty vector or a *CsNAC28*-GFP vector were introduced into tobacco leaves. The GFP fluorescence of the *CsNAC28*-GFP vector was detected in the nucleus, while the GFP signals of the empty vector were detected in the nucleus and cytoplasm (**Figure 6B**). The results are consistent with the predicted role of CsNAC28 as a transcription activator.

### *CsABF2* binds and activates *CsNAC28* expression

*CsABF2* was examined as a key transcription factor in regulating the tea cultivar’s drought tolerance (Lu et al., 2021). To verify whether the ABRE element on CsNAC promoters could be recognized and bonded by *CsABF2*, the 1388-bp length of the *CsNAC28* promoter was fused to the *Aureobasidin A* (AbA) gene to generate the *CsNAC28Pro-pABAi* vectors that confer resistance to AbA. Then, the linearized *CsNAC28Pro-pABAi* vector (digested by BstB I) was co-transformed with the *CsABF2*-pGAD vector and tested on SD/-Trp/150mM AbA media (**Figure 6C**). The result showed that CsABF2 could recognize and bind the *cis*-element in the promoter of *CsNAC28 in vitro*. Then, to explore the effect of *CsABF2* on *CsNAC28* transcription *in vivo*, we cloned the promoter regions of *CsNAC28* to fuse them into the LUC reporter vector. Compared with the control, the co-expression of *CsABF2* dramatically increased the promoter activity of *CsNAC28* by 3.6-fold (**Figure 6D**). Furthermore, we transiently silenced *CsABF2* in tea plants to investigate the relationship between *CsABF2* and *CsNAC28*. Compared with *CsABF2/sODN* tea plants, *CsABF2* and *CsNAC28* were decreased by 60% and 41%, respectively, in *CsABF2/AsODN* tea plants (**Figure 6E**). Altogether, the results suggested that *CsABF2* could recognize the ABRE element on the promoter of *CsNAC28* and activate the expression of *CsNAC28*.

### Overexpression of *CsNAC28* enhances drought tolerance in transgenic Arabidopsis

**Figure 7:**
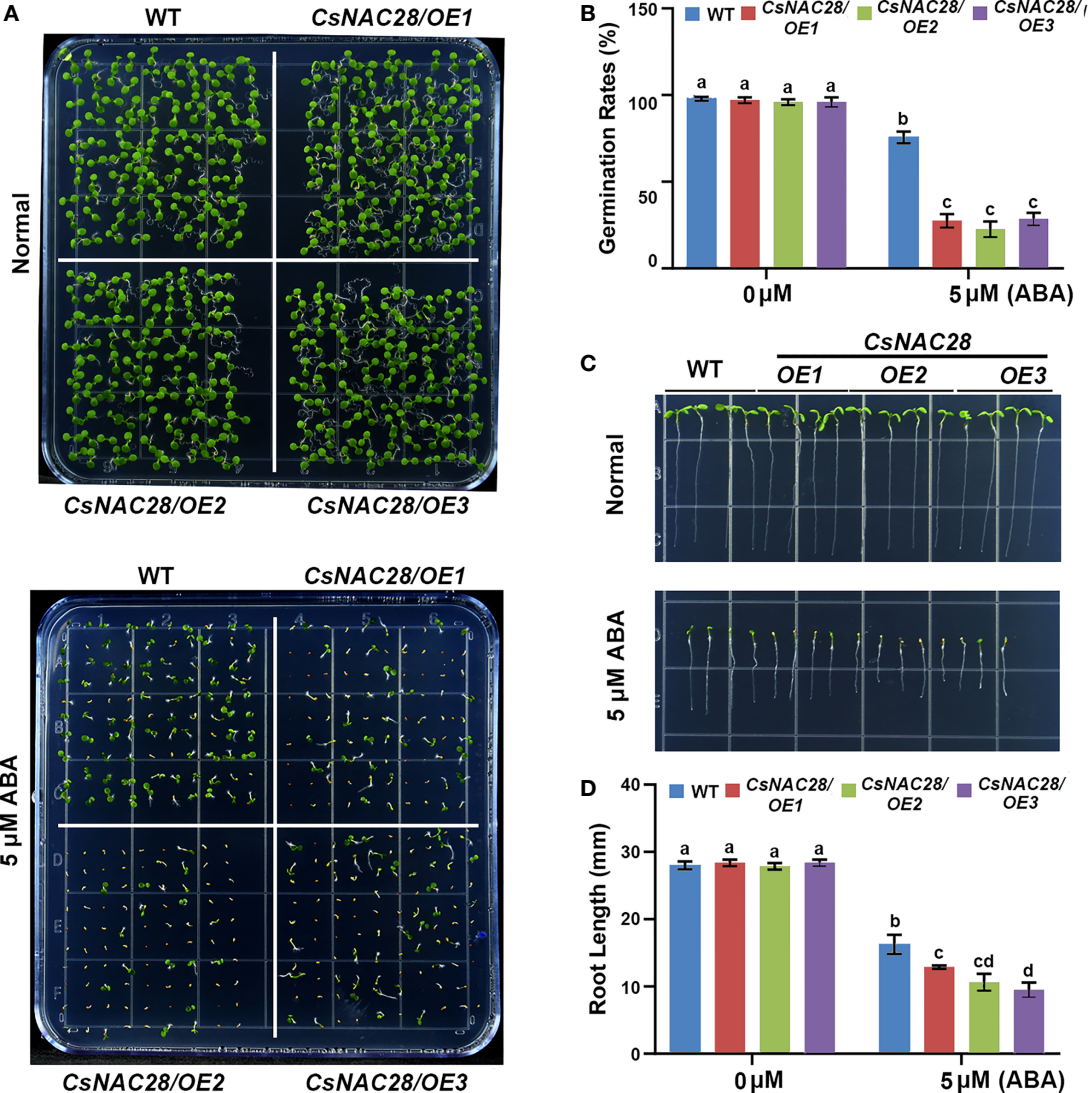
Overexpression of CsNAC28 increases ABA sensitivity in transgenic Arabidopsis. **(A)** Germination assays for each lines under different concentrations of ABA treatments. **(B)** Germination percentage of each line under different concentrations of ABA treatments. **(C)** Root length assays for different lines under different concentrations of ABA treatments. **(D)** Quantification of root length under different concentrations of ABA treatments.

The ABA signaling pathway is critical for plant adaptation to drought stress. When a plant is subjected to drought stress, ABA rapidly accumulates in the roots and leaves (Finkelstein et al., 2002; Kuromori et al., 2018). To explore the role of *CsNAC28* in the adaption to drought stress in plants, we heterogeneously expressed *CsNAC28* in Arabidopsis. Three overexpression lines with relatively higher expression levels were selected for further analysis (**Supplementary Figure S4**). Since the expression of *CsNAC28* was upregulated by ABA treatment, we spotted the seeds of the wild type (WT) and three *CsNAC28/OE* lines on 1/2MS medium with or without the addition of 5 μM ABA. As the results showed, no significant difference in growth phenotype and germination rate was observed in the WT and *CsNAC28/OE* transgenic lines on the 1/2MS medium. However, when grown on 1/2MS medium with 5 μM ABA added, three *CsNAC28/OE* lines had lower germination rates than the WT (**Figures 7A, B**) to different degrees. Moreover, the roots of the WT were longer than the roots of the three *CsNAC28/OE* lines (**Figures 7C, D**). Our data indicated that *CsNAC28* overexpression in Arabidopsis increased the sensitivity to ABA.

**Figure 8:**
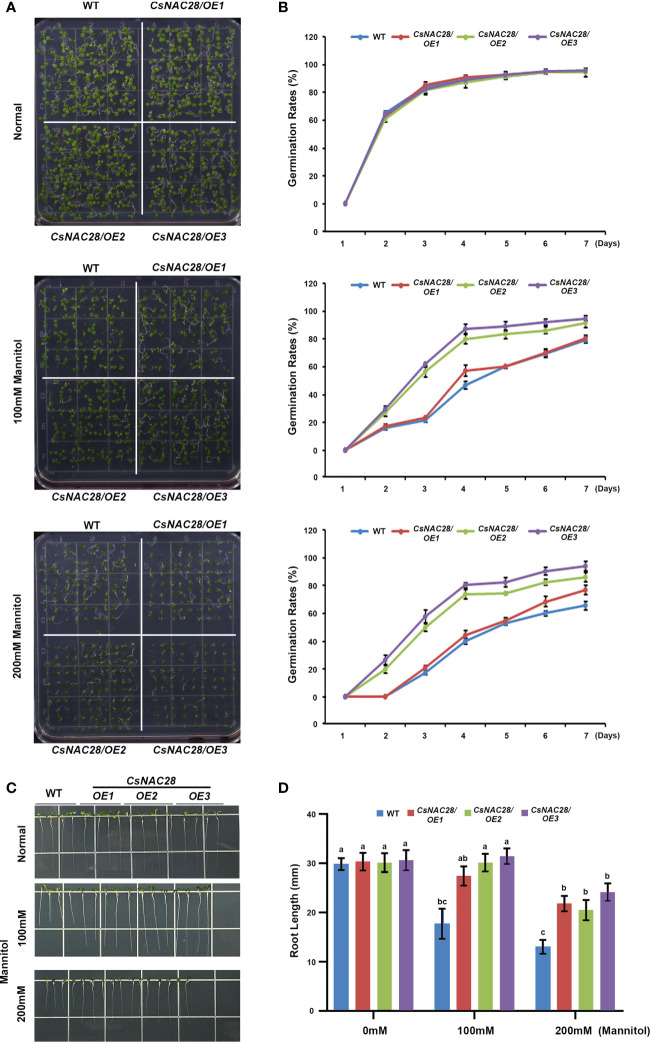
CsNAC28 overexpression promotes seed germination under simulated drought conditions. **(A)** Germination assay under different concentrations of mannitol treatments. **(B)** Germination percentage of each line after 7-day simulated drought treatment. **(C)** Root elongation assays under different concentrations of mannitol treatments. **(D)** Quantification of root length under different concentrations of mannitol treatments. Different letters indicate significant differences at P-value<0.05.

To further examine the drought tolerance of the *CsNAC28/OE* lines, mannitol treatment was used to simulate drought stress. Three *CsNAC28/OE* lines and the WT were sown on 1/2MS medium containing 0 mM, 100 mM, or 200 mM mannitol. Under normal growth conditions, there was no obvious difference in the germination rate (**Figures 8A, B**) or root length (**Figures 8C, D**) between the transgenic Arabidopsis and the WT. When grown on the 1/2MS medium plus 100 mM mannitol, the germination rate of the WT was 80% and the root growth was slightly impaired. Compared with the influence on the WT, the germination percentages of the three *CsNAC28/OE* lines were higher (84%, 92%, and 96%), and consistently, the root lengths were longer (**Figure 8**). When the concentration of mannitol increased to 200 mM, the germination rate of the WT fell to 65% and the root growth was severely retarded. In this condition, the *CsNAC28/OE* lines with higher germination rates (**Figures 8A, B**) and longer root lengths exhibited resistance to mannitol treatment (**Figures 8C, D**).

**Figure 9:**
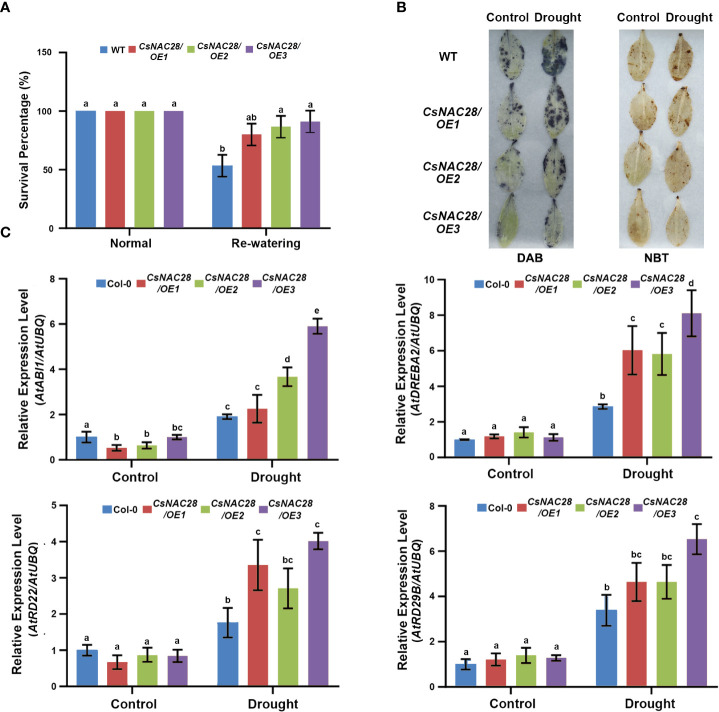
Overexpression of CsNAC28 improves drought stress tolerance in transgenic Arabidopsis. **(A)** Survival rates of the WT and transgenic lines at 7d of re-water. **(B)** Histochemical detection of hydrogen peroxide (H_2_O_2_) and superoxide (O^2−^) using DAB staining and NBT staining. **(C)** The relative expression of four abiotic stress-responsive genes in transgenic lines and the WT under normal and drought stress conditions. Error bars indicate the SD of three biological replicates.

Four-week-old seedlings were subjected to drought stress to further identify the function of *CsNAC28* in drought stress during the adult stage. The majority of the WT leaves were seriously wilted and unable to recover after re-watering or were dead. Only 53% of the WTs subjected to drought treatment survived. However, the transgenic lines (*CsNAC28/OE1, CsNAC28/OE2*, and *CsNAC28/OE3*) wilted only slightly after drought treatment and grew normally after re-watering; For them the final average survival percentages were 80%, 86%, and 91%, respectively (**Figure 9A**). In addition, we measured the hydrogen peroxide (H_2_O_2_) and O^2−^ content in leaves under normal and drought conditions using DAB and NBT staining to see whether those levels were associated with improved drought tolerance. Under normal conditions, the WT and three *CsNAC28/OE* lines had little staining. When exposed to drought stress, the staining of the WT leaves was substantially greater than of the leaves of the *CsNAC28/OE* lines (**Figure 9B**).

It is well known that *ABI1* and *DREB2A* are involved in ABA signaling transduction and that *RD22* (responsive to dehydration 22) and *RD29B* are marker genes in the ABA pathway (Yamaguchi and Shinozaki, 1993; Liu et al., 2020; Yu et al., 2021; Sun et al., 2022). To further explore the role of *CsNAC28* overexpression in drought response pathways, the expression of these genes was analyzed. Under normal conditions, there was no obvious difference in expression pattern between the *CsNAC28/OE* and the WT plants. Under drought conditions, however, the expression of all these genes was significantly induced. Furthermore, the expression levels were higher in the *CsNAC28/OE* plants than in the WT plants. These results demonstrated that *CsNAC28* overexpression resulted in the additional upregulation of drought-responsive genes during drought treatment (**Figure 9C**).

## Discussion

NAC transcription factors make up the most abundant plant-specific transcription family that plays a central role in plants’ growth, development, and response to environmental stimuli. Previous reports showed that 45 putative NAC sequences were identified in the tea plant (Wang et al., 2016b). In the current study, a total of 104 members of the enlarged NAC gene family were identified in *C. sinensis*. The genome sizes of Arabidopsis (125 Mb), *O. sativa* (480 Mb), and *P. trichocarpa* (1.07 Gb) are smaller than the genome size of *C. sinensis*, whereas the members of the NAC gene family of Arabidopsis (105), rice (151), and *P. trichocarpa* (167) are more numerous than in the tea plant (104) (Guillaume et al., 2003; Ooka et al., 2003; Tuskan et al., 2006; Hu et al., 2010; Korbinian et al., 2011; Nuruzzaman et al., 2010; Schneeberger et al., 2011). In particular, the CsNAC genes in subgroups B, F, and H are far fewer than in Arabidopsis and *P. trichocarpa*. The exon/intron location patterns of CsNACs were unified in all the phylogenetic subfamilies studied. Similar exon/intron structures were observed in the same subgroups as in Arabidopsis, *O. sativa*, and *P. trichocarpa* (Ooka et al., 2003; Hu et al., 2010; Nuruzzaman et al., 2010).

Genome duplication events have increased the evolution and expansion speed of these essential genes, which might provide genetic diversity and a greater ability for plants to survive under a variety of environmental pressures (Blanc et al., 2003; Crow et al., 2006). There are 49 segmental duplications found in the tea plants, and many species such as Arabidopsis, *O. sativa*, *P. trichocarpa*, and potatoes experienced an abundance of segmental duplication events (Olsen et al., 2005; Hu et al., 2006; Hu et al., 2010; Yan et al., 2021). Therefore, segment gene duplication plays a vital role in NAC gene family expansion. However, in comparing the size of the genome with NAC genes in different species, the low number of NAC genes in *C. sinensis* might be due to the whole-genome duplication events.

Drought stress is one of the most severe difficulties encountered by land plants; it disrupts plants’ metabolism, photosynthesis, and cell structure, thereby impairing plants’ productivity (Pandey and Shukla, 2015; Chen et al., 2017; Qing et al., 2022). Recently, many studies have shown that NACs can upgrade the drought resistance of Arabidopsis, rice, wheat, and maize (Tran et al., 2004; Hu et al., 2006; Huang et al., 2015; Zhu et al., 2016). However, the research on stress-related NAC genes in tea plants was limited. NAC genes operate at multiple levels in ABA signaling networks (Fujita et al., 2011; Kuromori et al., 2018). Many NAC transcription factors have been reported to be upregulated by exogenous ABA and involved in an ABA-dependent signaling pathway in response to drought stress (Fujita et al., 2004; Ding et al., 2019; Chen et al., 2021). Herein, we showed that the expression of *CsNAC28* was strongly induced by treatment with ABA and PEG. The fact that the peak values of relative expression were different between them may be due to the fact that exogenous ABA induces conduction faster than drought stress induces endogenous signaling. Previous works indicated that ABF could bind to the ABRE *cis*-acting element in the promoter of NAC and thereby became involved in a drought stress response (Jia et al., 2022). Our study found that *CsABF2* could bind to the ABRE *cis*-acting element in the promoter of *CsNAC28* and activate *CsNAC28* expression.

ABA is the phytohormone most closely related to drought stress responses in plants. It causes stomatal closure, which decreases water loss, and is thus critical for drought resistance (Wu et al., 2019). Overexpression of *CsNAC28* in Arabidopsis reduced germination rates and shortened root lengths when ABA medium was added. Our study supported the finding that *CsNAC28* overexpression in Arabidopsis resulted in increased sensitivity to ABA and promoted ABA-mediated stomatal closure, which helped plants conserve water and have improved survival rates. Overexpression of *CsNAC28* in Arabidopsis upregulated the expression of drought-responsive genes (*ABI1*, *DREB2A*, *RD22*, and *RD29B*) and enhanced plants’ survival rates under drought stress conditions. Our study found that *CsNAC28* was involved in the response to drought stress dependent on the ABA signal transduction pathway.

In contrast to the highly conserved NAC-binding domain at the N-terminal of NAC family proteins, the C-terminal transcription regulatory region is highly variable and usually functions as a transcriptional repressor or activator (Puranik et al., 2012). In this study, we demonstrated that *CsNAC28* was located in the nucleus and functioned as a transcriptional activator to modulate abiotic stress tolerance positively. Drought stress causes the excessive accumulation of ROS, which destroys plant performance and thus reduces crop yields. Hence, having an ROS scavenging system is crucial for plants to cope with drought stress (Verslues et al., 2006; Guo et al., 2022). The role of NAC transcription factors in regulating ROS scavenging systems under drought stress has been explored in other species. The Arabidopsis NAC transcription Jungbrunnen1 is induced by H_2_O_2_ and reduces the level of H_2_O_2_ in cells and improves various degrees of abiotic stress tolerance (Wu et al., 2012). In rice, *SNAC3* confers drought tolerance through the modulation of ROS (Fang et al., 2015). We found that compared with WT plants, *CsNAC28/OE* plants had a significantly reduced ROS content under drought stress. These findings indicated that WT plants suffered more serious oxidative damage than *CsNAC28* transgenic plants during the drought stress response. The improved antioxidant capability of *CsNAC28/OE* plants enhanced the drought resistance of transgenic Arabidopsis at the cellular level, laying the foundation for its drought-tolerant phenotype.

## Conclusion

NAC plays an essential role in responses to abiotic stresses. Herein, we have identified 104 NAC transcription factors in *C. sinensis* and presented a comprehensive analysis of them. Importantly, our findings suggested that *CsNAC28* contributes to drought tolerance by regulating the expression of ABA-related genes and the antioxidant system. These results provide physiological and molecular evidence for the participation of *CsNAC28* in plants’ drought tolerance.

## Data availability statement

The original contributions presented in the study are included in the article/**Supplementary Material**. Further inquiries can be directed to the corresponding author.

## Author contributions

XZ and GH designed the research and performed most of the experiments. LL, ZL, DL, YH, YZ, HT, JW and QL performed some of the experiments. XZ and GH were responsible for data analysis and wrote the manuscript. The authors agree with the content of the manuscript. All authors contributed to the article and approved the submitted version.

## Funding

This work was funded by the National Natural Science Foundation of China under Grant No. 32000234, and the Zhejiang Provincial Natural Science Foundation of China under Grant No. LR22C020003, the Major Science and Technology Special Project of Variety Breeding of Zhejiang Province (2021C02067-7), and the State Key Laboratory for Managing Biotic and Chemical Threats to the Quality and Safety of Agro-products under Grant No. 2021DG700024-KF202102.

## Conflict of interest

The authors declare that the research was conducted in the absence of any commercial or financial relationships that could be construed as a potential conflict of interest.

## Publisher’s note

All claims expressed in this article are solely those of the authors and do not necessarily represent those of their affiliated organizations, or those of the publisher, the editors and the reviewers. Any product that may be evaluated in this article, or claim that may be made by its manufacturer, is not guaranteed or endorsed by the publisher.
