# Corrigendum: Genome-wide characterization of NAC transcription factors in *Camellia sinensis* and the involvement of CsNAC28 in drought tolerance

**DOI:** 10.3389/fpls.2023.1289503

**Published:** 2023-10-04

**Authors:** Xueying Zhang, Linying Li, Zhuoliang Lang, Da Li, Yuqing He, Yao Zhao, Han Tao, Jiqian Wei, Qingsheng Li, Gaojie Hong

**Affiliations:** ^1^ Key Laboratory for Managing Biotic and Chemical Threats to the Quality and Safety of Agro-products, Key Laboratory of Biotechnology in Plant Protection of Ministry of Agriculture and Rural Affairs, Key Laboratory of Biotechnology in Plant Protection of Zhejiang Province, Institute of Virology and Biotechnology, Zhejiang Academy of Agricultural Sciences, Hangzhou, China; ^2^ College of Advanced Agricultural Sciences, Zhejiang A&F University, Hangzhou, China; ^3^ Institute of Sericulture and Tea, Zhejiang Academy of Agricultural Sciences, Hangzhou, China; ^4^ Ecology and Energy Section, Hangzhou Agricultural Technology Extension Center, Hangzhou, China

**Keywords:** *Camellia sinensis*, expression pattern, NAC transcription factor, drought stress, abscisic acid

In the published article, there was an error in the **Funding** statement. The grant number for funding from the Zhejiang Provincial Natural Science Foundation of China was incorrectly reported as “No. LR22C02003”. The correct **Funding** statement appears below.

“This work was funded by the National Natural Science Foundation of China under Grant No. 32000234, and the Zhejiang Provincial Natural Science Foundation of China under Grant No. LR22C020003, the Major Science and Technology Special Project of Variety Breeding of Zhejiang Province (2021C02067-7), and the State Key Laboratory for Managing Biotic and Chemical Threats to the Quality and Safety of Agro-products under Grant No. 2021DG700024-KF202102”.

The authors apologize for this error and state that this does not change the scientific conclusions of the article in any way. The original article has been updated.

