# Supplementary material for: Genome-wide characterization of NAC transcription factors in Camellia sinensis and the involvement of CsNAC28 in drought tolerance
Source: Front Plant Sci. 2022 Nov 24;13:1065261. doi: 10.3389/fpls.2022.1065261 (PMC9731689; doi:10.3389/fpls.2022.1065261)
Supplement: Supplementary file 3 [file DataSheet_1.pdf]

Genome-wide Characterization of NAC transcription factors in *Camellia sinensis* and the involvement of CsNAC28 in drought tolerance

Xueying Zhang[1], Linying Li[1], Zhuoliang Lang[1, 3], Da Li[2], Yuqing He[1], Yao Zhao[1], Han Tao[1], Qingsheng Li[2], Gaojie Hong[1,*]

[1] Key Laboratory for Managing Biotic and Chemical Threats to the Quality and Safety of Agro-products, Key Laboratory of Biotechnology in Plant Protection of Ministry of Agriculture and Rural Affairs, Key Laboratory of Biotechnology in Plant Protection of Zhejiang Province, Institute of Virology and Biotechnology, Zhejiang Academy of Agricultural Sciences, 198 Shiqiao Road, Hangzhou 310021, China

[2] Institute of Sericulture and Tea, Zhejiang Academy of Agricultural Sciences, Hangzhou, China

[3] College of Advanced Agricultural Sciences, Zhejiang A&F University, Hangzhou, China

* Corresponding author

Author for correspondence:

Gao-Jie Hong

Tel: +86-571-86419021

E-mail: gjhong@126.com

Supplemental Figures

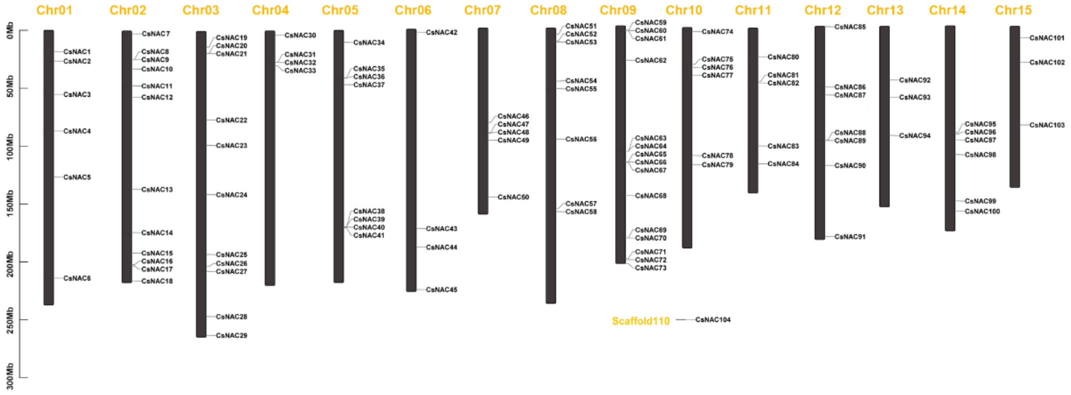

Supplemental Figure S1| Distribution of 104 CsNAC genes on 15 chromosomes.

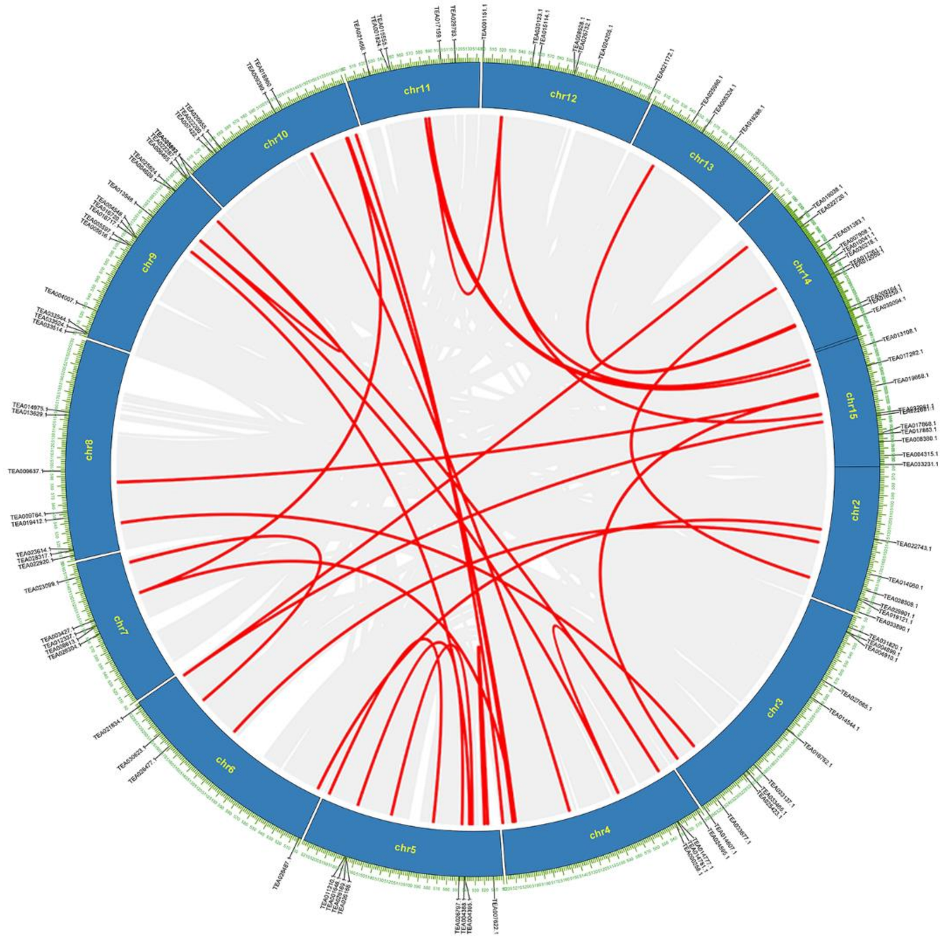

Supplemental Figure S2| Schematic representations of the interchromosomal relationships of CsNAC genes. Red lines indicate segmental duplicates of NAC gene pairs.

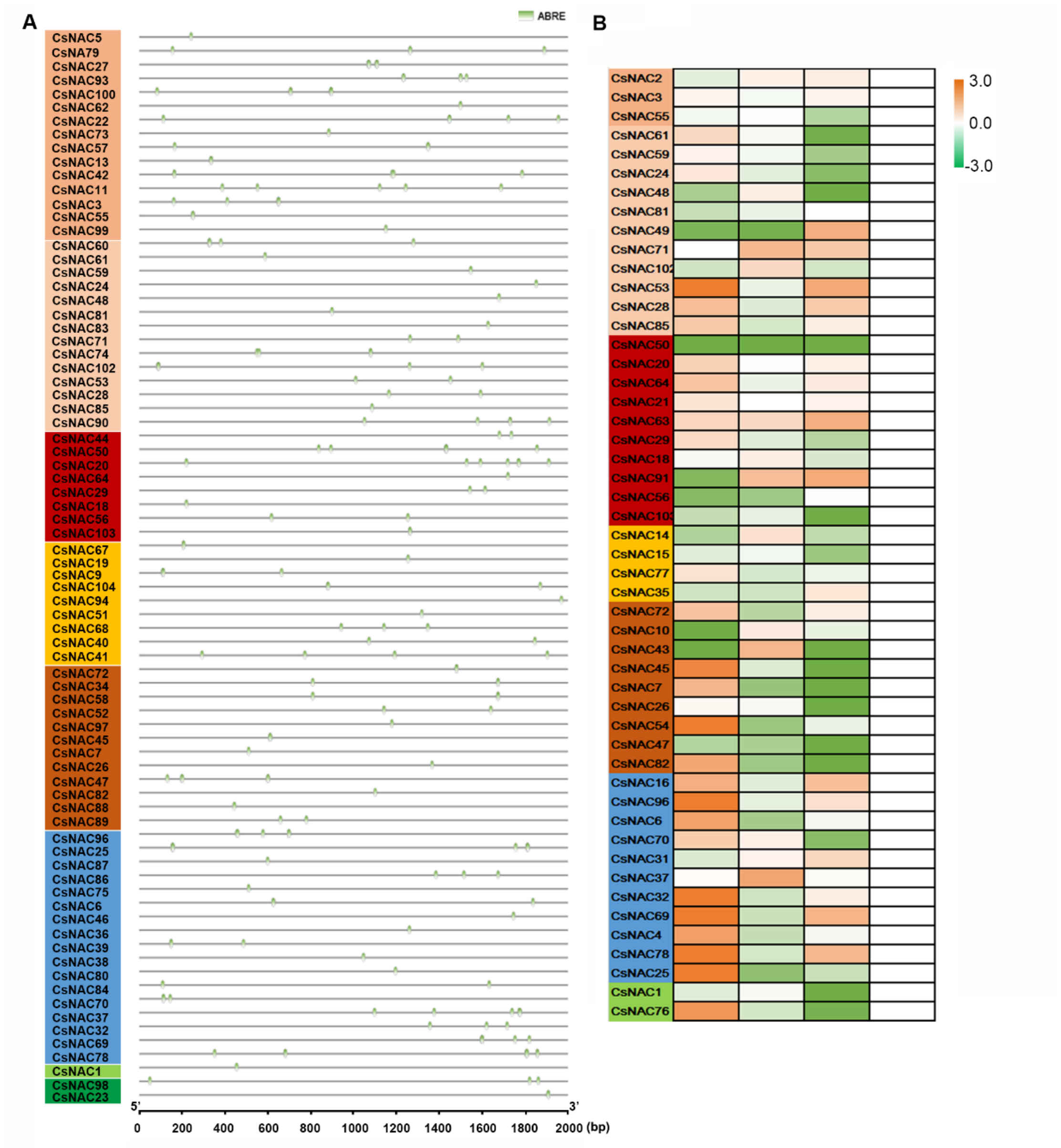

Supplemental Figure S3| AREB *cis*-acting elements of CsNAC gene promoter regions. A Distribution of ABRE cis-element in promoter of CsNAC. **B** Expression pattern of CsNACs in response to drought stress.

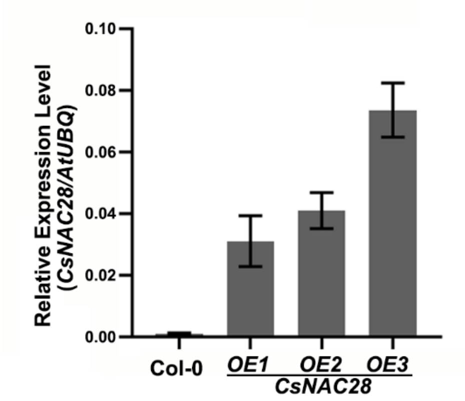

Supplemental Figure S4| Gene expression of *CsNAC28 in* Col-0 and over-expressed plants.
